# Correction: On the relationship between predictive coding and backpropagation

**DOI:** 10.1371/journal.pone.0320944

**Published:** 2025-03-25

**Authors:** Robert Rosenbaum

In the subsection titled “A strict interpretation of predictive coding does not accurately compute gradients” within the Results section, there is an error in the sixth sentence of the seventh paragraph. The correct sentence is: Note that the prediction errors, $\in_{l}=f_{l}\left(v_{l-1};\theta_{l}\right)-v_{l}$, should also be updated on each iteration.

There is an error in Algorithm 2 within the Results section. The equation $\in_{l}=v_{l}-f_{l}\left(v_{l-1};\theta_{l}\right)$ should be $\in_{l}=f_{l}\left(v_{l-1};\theta_{l}\right)-v_{l}$ and the equation $dv_{l}=-\in_{l}+\in_{l+1}\frac{\partial f_{l+1}\left(v_{l};\theta_{l+1}\right)}{\partial v_{l}}$ should be $dv_{l}=\in_{l}-\in_{l+1}\frac{\partial f_{l+1}\left(v_{l};\theta_{l+1}\right)}{\partial v_{l}}$.

There is an error in Algorithm 3 within the Results section. The equation $\in_{l}=v_{l}-{\hat{v}}_{l}$ should have been $\in_{l}={\hat{v}}_{l}-v_{l}$.

In the subsection titled “Predictive coding modified by the fixed prediction assumption using a step size of η = 1 computes exact gradients in a fixed number of steps” within the Results section, the equation $\in_{l}^{i}=v_{l}^{i-1}-{\hat{v}}_{l}$ in the third sentence of the third paragraph should have been $\in_{l}^{i}={\hat{v}}_{l}-v_{l}^{i-1}$.

In the subsection titled “Predictive coding modified by the fixed prediction assumption using a step size of η = 1 computes exact gradients in a fixed number of steps” within the Results section, the equation $\in_{l}^{i+1}-\in_{l}^{i}$ in the eighth sentence should be $\in_{l}^{i}-\in_{l}^{i+1}$.

In the subsection titled “Predictive coding with the fixed prediction assumption and η = 1 is functionally equivalent to a direct implementation of backpropagation” within the Results section, there is an error in the third sentence of the third paragraph. The correct sentence is: If we additionally want to compute the fixed point beliefs, then they can still be computed using the relationship $v_{l}={\hat{v}}_{l}-\in_{l}$.
